# Harnessing the power of ionising radiation to enhance cancer immunotherapy

**DOI:** 10.1002/ctm2.70307

**Published:** 2025-04-23

**Authors:** Kai Pang, Zhongtao Zhang

**Affiliations:** ^1^ Department of General Surgery Beijing Friendship Hospital Capital Medical University Beijing China

## 1 RADIATION TO WARM‐UP COLD TUMOURS

In resectable rectal cancer, pre‐operative chemoradiation therapy (CRT) significantly increases the amount of tumour‐infiltrated CD8^+^ T cell.^1^ And evidences from colorectal cancer mouse models suggest that the extent of CD8^+^ T‐cell infiltration is increased by tumour‐localised ionising radiation alone, without effect of chemotherapeutic agents.^2^ These are all concordant with current evidences and consensuses supporting the immune‐stimulating effect of radiotherapy. On the other hand, the extent of CD8^+^ T‐cell infiltration is a primary metric to distinguish between cold tumours and hot ones. And CD8^+^ T cell are also the primary effector of immune checkpoint inhibitor (ICI). Hence, from a theoretical standpoint, radiotherapy primes the micro‐environment of rectal cancer and lays a foundation for the efficacy of ICI.

In real‐world clinical context, results of our POLARSTAR trial do show that combining ICI with radiotherapy leads to enhanced tumour regression for resectable rectal cancer,^3^ and similar evidences are also acquired by other teams for resectable non‐small‐cell lung cancer (NSCLC).^4^ Moreover, long‐term results from the PACIFIC trial demonstrated that adding ICI sequentially after radiotherapy substantially increases 5‐year disease‐free survival and 5‐year overall survival for NSCLC,^5^ also confirming the synergism between radiation and ICI. Although results from mouse models point out a pan‐cancer nature of the synergistic effect,^6^ but several randomised controlled trials (RCTs) from other tumour types beyond rectal cancer and NSCLC failed to display improved efficacy for combining radiotherapy with ICI, mostly due to irrational sequence (i.e., starting ICI treatment before radiotherapy) and heavy interference from chemotherapy (i.e., chemoradiation plan containing heavy chemo content, with strong chemotherapy inhibiting circulating lymphocytes), highlighting the importance of pre‐investigation and comprehensiveness when designing an RCT.

## 2 INSIGHTS FROM POLARSTAR TRIAL

The POLARSTAR trial is part of our endeavor to explore the synergy between clinical radiotherapy and ICI in mismatch‐repair proficient (pMMR) solid tumours. It enrolled resectable (i.e., non‐metastatic) rectal cancer patients and treated them with standard‐of‐care CRT before surgery for all three groups (two experiment groups and a control group). Additionally, PD1 blockade was added to CRT in the two experiment groups, with PD1 blockade starting at different time points relative to CRT.^3^ The CRT was composed of 50 Gy X‐ray volumetric modulated arc therapy given in 25 fractions, accompanied by light dosage of oral chemo drug. We chose this pre‐operative treatment modality for the trial among all modalities of rectal cancer for the fact that it is composed primarily of radiotherapy and has the slightest chemotherapy content. And for ethical concerns we also did not modify the CRT schedule to completely remove the chemotherapy when designing the trial.

Results of the POLARSTAR trial demonstrated that the pathological complete response (pCR) rates for pMMR rectal cancer were 17.0% with radiotherapy alone and 34.0% with radiotherapy plus ICI. Ideally, the trial should also be designed with an additional group of patients receiving ICI alone (i.e., without radiotherapy). But the renowned NICHE trial have reported that pre‐operative ICI alone resulted in pCR rates of 5.6% for pMMR resectable colorectal cancer patients and 57.1% for mismatch‐repair deficient (dMMR) ones, confirming a lack of efficacy of ICI in pMMR patients while providing the POLARSTAR trial with a perfectly needed external reference.^7^ In this case, setting an additional control group of patients receiving pre‐operative ICI alone for the POLARSTAR trial is no longer ethically appropriate, nor necessary.

**FIGURE 1:**
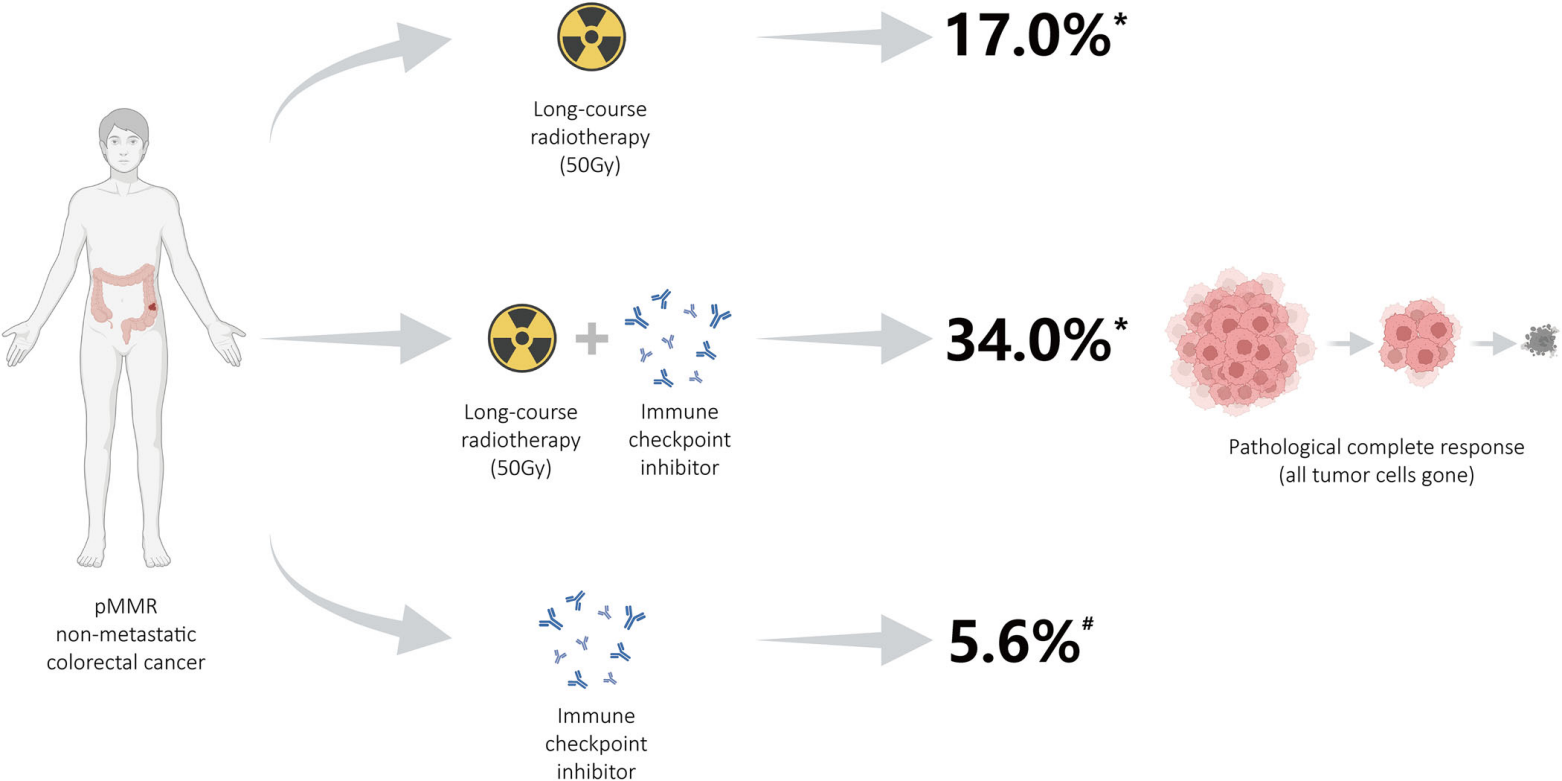
Synergism between clinical radiotherapy and immune checkpoint blockade is confirmed by substantially increased pathological complete response rate. ^*^Data from the POLARSTAR trial. ^#^Related data from the NICHE trial that can be used as an appropriate external reference.

As shown in Figure 1, results of our trial, together with the external reference from the NICHE trial, provided a vivid description on the synergistic effect between clinical radiotherapy and ICI in resectable pMMR colorectal cancer.

## 3 APPLICATIONS OF RATIONALE IN CLINICAL SETTING

**FIGURE 2:**
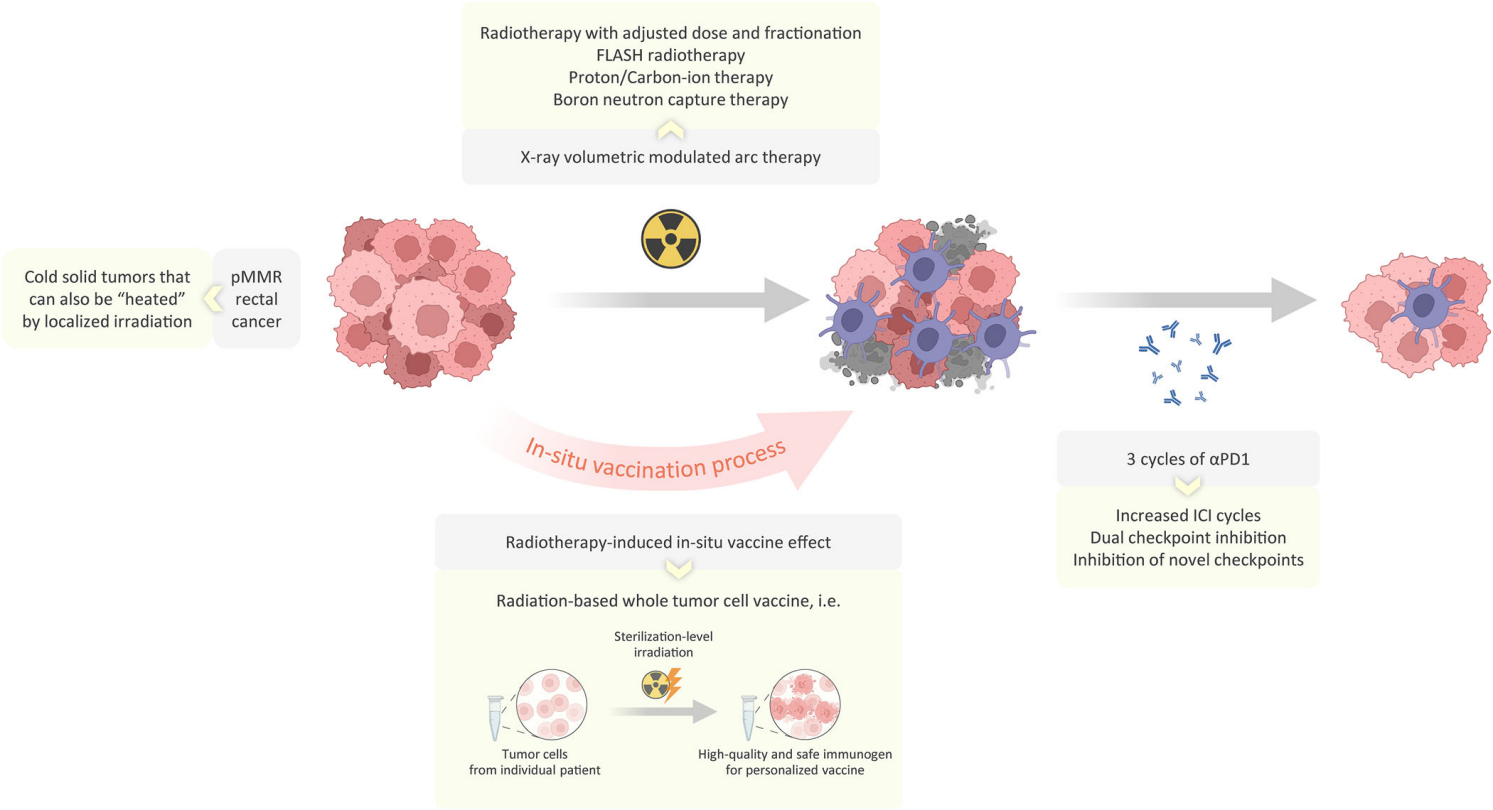
Further applications of the radiation plus immune checkpoint inhibitor (ICI) rationale.

The POLARSTAR trial is inspiring for the further application of combining radiotherapy and ICI in cold solid tumours (Figure 2), as rectal cancers are predominantly pMMR or micro‐satellite stable (over 95% in proportion), which is also the case for NSCLC and almost all other cancer types.^8^ Theoretically, if a certain type of tumour can be generally ‘heated up’ (i.e., CD8^+^ T‐cell infiltration enhanced) by localised irradiation, then the combination of radiation plus ICI would very probably lead to significant clinical benefit. But in real‐world clinical setting, evidences from randomised trials are still needed for wider application of the rationale in other tumour types beyond rectal cancer and NSCLC. Also, special considerations should be given to cold solid tumours with naturally inhibitory micro‐environment that perversely resist the infiltration of CD8^+^ T cells, such as liver cancer and pancreatic cancer.

The radiotherapy technique used in the POLARSTAR trial is X‐ray volumetric modulated arc therapy, which is representative of the current routine technique for long‐course radiotherapy of rectal cancer in China. Yet, the positive findings of the POLARSTAR trial implies that there's still potential for optimising the dose and fractionation of radiotherapy, so as to better facilitate checkpoint immunotherapy and maximise overall efficacy. Furthermore, novel radiotherapy techniques such as FLASH radiotherapy, proton/heavy‐ion therapy and boron neutron capture therapy are capable of delivering much more intensified dosage to tumour tissues to induce immunogenic tumour cell death in a presumably much more efficient manner with milder influence on healthy tissues, which, therefore, harbours exciting possibilities for the broader application of the radiation plus ICI approach.

On the other hand, there is also room for optimisation on the ICI part. In the renowned trial by Cercek et al., a 100% (12/12) complete response rate was achieved for resectable dMMR rectal cancer patients with single‐agent αPD1, where the duration of αPD1 treatment is exceptionally long (i.e., 6 months).^9^ This reminds us that three cycles of αPD1 following radiotherapy, as in the POLARSTAR trial, might be insufficient. Better efficacy seems very likely if we could increase the duration of αPD1 treatment, or opt for dual checkpoint inhibition (e.g., αPD1 plus αCTLA4). However, caution should be given to potential immune‐related adverse reaction (IRAR) when tailoring the combination, as some IRARs are hardly reversible (e.g., ICI‐related thyroiditis/myositis, etc.), despite generally low rate of occurrence according to results from the POLARSTAR trial.^3^

In terms of molecular mechanism, radiotherapy induces immunogenic tumour cell death, releasing inflammatory cytokines, adjuvant‐like molecules and tumour antigens to enhance adaptive anti‐tumour immunity. This process highly resembles the work of vaccines and is therefore termed the in situ vaccine effect of radiotherapy. In this context, our team examined the vaccine effect of sterilisation‐level irradiated autologous tumour cells on syngeneic mouse tumour models. Based on our unpublished results, we hypothesise that sterilisation‐level irradiated autologous tumour cells, together with appropriate artificial adjuvant, might constitute high‐quality, low‐cost and absolutely safe tumour vaccines, potentially avoiding the expensive and laborious process of neoantigen prediction.

In summary, radiation plus ICI represents a promising approach for the treatment of cold tumours, which constitute the predominant majority for almost all tumour types. Well‐designed translational researches and randomised clinical trials in this direction are essential for the clinical benefit of a broader patient community.

## AUTHOR CONTRIBUTIONS

*Conceptualisation*, *data curation and formal analysis*: Kai Pang. *Investigation*: Kai Pang and Zhongtao Zhang. *Methodology*: Kai Pang. *Project administration*: Kai Pang and Zhongtao Zhang. *Software*: Kai Pang. *Supervision*: Zhongtao Zhang. *Visualisation and writing—original draft*: Kai Pang. *Writing—review and editing*: Kai Pang and Zhongtao Zhang.

## CONFLICT OF INTEREST STATEMENT

The authors declare they have no conflicts of interest.

## FUNDING INFORMATION

The authors received no specific funding for this work.

## Data Availability

The data that support the findings of this study are available from the corresponding author upon reasonable request.
